# DSSS Signal Detection Based on CNN

**DOI:** 10.3390/s23156691

**Published:** 2023-07-26

**Authors:** Han-Qing Gu, Xia-Xia Liu, Lu Xu, Yi-Jia Zhang, Zhe-Ming Lu

**Affiliations:** 1 School of Information Science and Engineering, Zhejiang Sci-Tech University, Hangzhou 310018, China; hanqinggu@163.com (H.-Q.G.); xlhit@126.com (X.-X.L.); xulu@zstu.edu.cn (L.X.); 2 School of Aeronautics and Astronautics, Zhejiang University, Hangzhou 310027, China; zheminglu@zju.edu.cn

**Keywords:** direct sequence spread spectrum (DSSS), convolutional neural network (CNN), deep learning, DSSS signal detection, autocorrelation detection method, spread spectrum signal detection method

## Abstract

With the wide application of direct sequence spread spectrum (DSSS) signals, the comprehensive performance of DSSS communication systems has been continuously improved, making the electronic reconnaissance link in communication countermeasures more difficult. Electronic reconnaissance technology, as the fundamental means of modern electronic warfare, mainly includes signal detection, recognition, and parameter estimation. At present, research on DSSS detection algorithms is mostly based on the correlation characteristics of DSSS signals, and autocorrelation algorithm is the most mature and widely used method in practical engineering. With the continuous development of deep learning, deep-learning-based methods have gradually been introduced to replace traditional algorithms in the field of signal processing. This paper proposes a spread spectrum signal detection method based on convolutional neural network (CNN). Through experimental analysis, the detection performance of the CNN model proposed in this paper on DSSS signals in various situations has been compared and analyzed with traditional autocorrelation detection methods for different signal-to-noise ratios. The experiments verified the estimation performance of the model in this paper under different signal-to-noise ratios, different spreading code lengths, different spreading code types, and different modulation methods and compared it with the autocorrelation detection algorithm. It was found that the detection performance of the model in this paper was higher than that of the autocorrelation detection method, and the overall performance was improved by 4 dB.

## 1. Introduction

With the continuous development of technology, wireless communication technology has become increasingly mature and widely used in various communication systems in daily life. At the same time, in the rapidly developing modern military field of information technology, in order to enhance the concealment, security, and anti-interference ability of wireless communication, spread spectrum communication technology has also been widely applied in military communication systems [1]. Spread spectrum communication is a communication method that uses pseudo-random sequences to modulate information code sequences and broaden their spectrum. According to Shannon’s theorem, spread spectrum communication reduces the power spectrum of the signal during the process of expanding the spectrum, making the communication signal more covert. Direct sequence spread spectrum (DSSS) communication is one of the most widely used spread spectrum communication methods. It modulates information code through a high-speed pseudo-code sequence to the spread spectrum so that the signal energy is greatly reduced such that it is completely submerged by noise. It has the advantages of good concealment, relatively low power spectral density, strong anti-interception ability, etc., and is widely used in various civil and military communication systems. There has been research on the application of spread spectrum signals in the field of 5G communication [2,3].

In modern warfare, electronic warfare has gradually become an important factor affecting the process and outcome of warfare. The dominant party in electronic warfare will have a higher chance of winning and can gain more strategic advantages during the combat process, which can better suppress the disadvantaged party [4,5]. With the wide application of DSSS signals, the comprehensive performance of DSSS communication systems has been continuously improved, making the electronic reconnaissance link in communication countermeasures more difficult. Electronic reconnaissance technology, as the fundamental means of modern electronic warfare, mainly includes signal detection, recognition, and parameter estimation. In non-cooperative situations [6], if the intercepted signal cannot be identified and the parameter estimated, it will be impossible to demodulate the intercepted signal and obtain useful information. In order to more effectively monitor and interfere with enemy signals, it is particularly important to accurately and efficiently identify the intercepted signals and estimate their parameters in the increasingly complex electromagnetic environment. Therefore, in non-cooperative situations, it is of great practical significance to study how to accurately detect direct spread spectrum signals in real time and estimate their parameters.

DSSS detection is the foundation of parameter estimation and demodulation of direct spread spectrum signals, and is an important research topic in the field of communication reconnaissance [7]. The traditional detection methods for direct spread spectrum signals mainly include energy detection, correlation detection, high-order statistics detection, and cyclic spectrum detection.

The energy detection method was first developed by Urkowitz [8]. Its main basis is the energy of the signal and noise, as well as the energy greater than the noise. By setting an appropriate threshold, the presence of a signal can be detected. However, if the noise power is too high and the threshold value is uncertain, it will lead to a decrease in algorithm performance.

The correlation detection method is a detection algorithm of direct sequence spread spectrum signal based on the difference in autocorrelation power spectral density between direct sequence spread spectrum signal and noise. Javed et al. proposed a detection method for direct spread spectrum signals in multi-signal environments based on autocorrelation fluctuations [9]. This method is designed for situations where the received signal includes not only the direct spread spectrum signal but also other effective signals with high signal-to-noise ratios. It mainly improves the detection performance of the direct spread spectrum signal by suppressing the influence of the main peak value on the threshold value, thereby reducing the influence of other signals on blind detection of the direct spread spectrum signal. Based on previous research, Zhang et al. proposed a detection method combining wavelet decomposition and delayed correlation to address the problem of discontinuous and unstable correlation peaks in DSSS signals under low signal-to-noise ratio conditions [10]. The noise of the signal is reduced through wavelet decomposition, making the correlation peaks of the DSSS signal more obvious and easier to detect, effectively improving the detection performance of the direct spread spectrum signal in low signal-to-noise ratio situations.

The high-order statistics detection method mainly detects signals through different characteristics of high-order statistics. High-order cumulant, high-order moment, high-order moment spectrum, and high-order cumulant spectrum are the four most common high-order statistics. The high-order cumulant detection method was first applied to the detection of DSSS signals proposed by Spooner and Gardner [11]. On this basis, Zhang and Zhang proposed an improved fourth-order statistics method based on 1-D slice and adaptive linear filter to realize the detection of DSSS signals [12], effectively reducing the computational complexity of the algorithm. On the basis of previous studies, Shi et al. studied the fourth-order cumulant of Unbalanced Quadrature Phase Shift Keying (UQPSK)-DSSS signals and proposed a detection method for UQPSK-DSSS signals based on the fourth-order cumulant slice [13].

The main basis of the cyclic spectrum detection method is that the direct spread spectrum signal has cyclostationary characteristics while the noise signal does not, and the cyclic spectrum has the characteristics of suppressing noise and interference. Therefore, the cyclic spectrum detection algorithm is suitable for signal detection under strong noise conditions. Some researchers proposed the improved cyclic spectrum detection algorithm based on data segmentation and overlap preservation processing [6]. The algorithm is mainly to overlap and segment the received signal, then model whether there is a DSSS signal as a binary hypothesis problem according to the characteristics of the cyclic spectral density function, and finally complete the detection of the DSSS signal through a cyclic spectrum detector, without prior knowledge. The statistics of the cyclic spectrum detector include cyclic spectrum amplitude. Other researchers proposed the improved cyclic spectrum detection method based on set averaging to address the issue of performance degradation in cyclic spectrum detection under limited data conditions [14]. This method achieved direct spread spectrum signal detection under typical interference conditions, such as low signal-to-noise ratio and single-tone interference and narrowband interference.

With the emergence and development of artificial intelligence technology, neural network technology is gradually being applied in the research of direct spread spectrum signal detection [15]. Some researchers proposed the phased detection method based on cyclostationary characteristics for the detection of direct spread spectrum ultra-wideband signals under low signal-to-noise ratio conditions [16]. This method first uses the energy detection method for detection and then uses the cyclic spectrum method for detection if the direct spread spectrum signal cannot be detected. Different from traditional cyclic spectrum detection, this method converts the three-dimensional cyclic spectrum of signal and noise into Grayscale. According to the difference between the two, a convolutional neural network is used to train the input image, extract features, and then detect the direct spread spectrum signal through the trained network. Wei et al. proposed a deep-learning-based direct spread spectrum signal detection method that does not require the conversion of signals into images [17]. This method does not require manual feature extraction in advance, and directly sends the direct spread spectrum signal and noise signal into the convolutional neural network (CNN) for training. On this basis, they also proposed a hybrid detection method based on CNN CORR, which uses the autocorrelation results of the signal to replace the signal and train it into the CNN to reduce computational complexity. Experiments have shown that the detection performance of this method is significantly better than traditional autocorrelation algorithms.

From above, we can see that current research on direct spread spectrum signal detection algorithms is mostly based on the correlation characteristics of DSSS signals, and autocorrelation algorithm is the most mature and widely used method in practical engineering. With the continuous development of deep learning, deep learning methods have gradually been introduced to replace traditional algorithms in the field of signal processing. This paper proposes a spread spectrum signal detection method based on CNN. Through experimental analysis, the detection performance of the CNN model proposed in this paper on DSSS signals in various situations has been compared and analyzed with traditional autocorrelation detection methods for different signal-to-noise ratios.

This paper first briefly explains the BPSK-DSSS signal model and the detection theory of DSSS signals. Then, a detailed introduction was provided for the CNN model and its parameters proposed in this article, and a reasoning analysis was conducted on the preprocessing before data input into the CNN and the important operations that need to be performed after input into the network. Then, a brief introduction was provided for the generation of the dataset and the training process of the model in the experiment. Finally, the estimation performance of the model in this paper was verified through experiments under different signal-to-noise ratios, different spreading code lengths, different spreading code types, and different modulation methods. A comparative analysis was conducted with the autocorrelation detection algorithm in the end.

## 2. DSSS Signal Model and Detection Theory

### 2.1. BPSK-DSSS Signal Model

**Figure 1:**
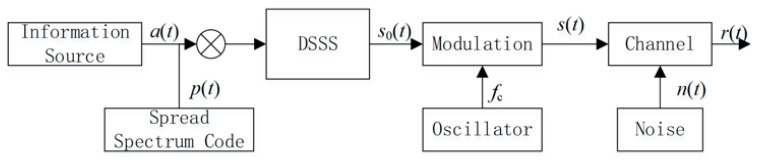
Block diagram of DSSS generation.

The generation of Binary Phase Shift Keying (BPSK)-modulated direct sequence spread spectrum signal is shown in Figure 1.

r(t)=s(t)+n(t) refers to direct sequence spread spectrum signal containing noise, s(t) refers to pure direct sequence spread spectrum signal, and n(t) refers to Gaussian white noise signal with mean value of zero and variance of $\sigma_{n}^{2}$. The formation process of s(t) is as follows: $s_{0}\left(t\right)$ is obtained by multiplying the original information sequence a(t) with the Pseudo-Noise (PN) code sequence p(t) directly and then modulating $s_{0}\left(t\right)$ with BPSK to obtain s(t), where $s_{0}\left(t\right)$ is the baseband signal after spreading. The specific definition of s(t) is shown in Equation (1) [17].

(1) $$s\left(t\right)=Aa\left(t\right)p\left(t\right)\cos\left(2\pi f_{c}t\right)$$

Among them, *A* represents amplitude, $f_{c}$ represents carrier frequency, and a(t) and p(t) are shown in Equations (2) and (3).

(2) $$a\left(t\right)=\sum_{j=-\infty }^{+\infty }a_{j}g\left(t-jT_{a}\right)$$

(3) $$p\left(t\right)=\sum_{k=-\infty }^{+\infty }p_{k}g\left(t-kT_{p}\right)$$

where $a_{j}\in \left\{-1,+1\right\}$ represents the original information code, $p_{k}\in \left\{-1,+1\right\}$ represents the PN code, g(t) represents the gate function, $T_{a}$ represents the width of the information code, and $T_{p}$ represents the width of the spread spectrum code.

If the PN code sequence of one cycle is denoted as $h\left(t\right)=\sum_{i=1}^{N}c_{i}g\left(t-iT_{p}\right)$, then p(t) can also be expressed as Equation (4).

(4) $$p\left(t\right)=\sum_{k=-\infty }^{+\infty }h\left(t-kT_{a}\right)$$

Among them, $T_{p}=T_{a}/N$, *N* is the length of the spread spectrum code. In this paper, the values of *N* are 127,255,511,1023,2047.

### 2.2. Mathematical Model for DSSS Signal Detection

In non-cooperative communication, detecting whether the received signal is a DSSS signal can be modeled as a binary hypothesis test problem. Assuming $H_{0}$ represents only noisy signals and $H_{1}$ is a DSSS signal with noise, we can mathematically express it as Equation (5).

(5) $$\begin{array}{l} H_{0}:r(k)=n(k)\\ H_{1}:r\left(k\right)=s\left(k\right)e^{j(2\pi k\Delta f+\Delta \theta )}+n\left(k\right) \end{array}$$

Among them, r(k) represents the sampled received signal, s(k) represents the sampled sequence, and n(k) represents the sampled sequence of Gaussian white noise signal, with a mean of 0 and a variance of $\sigma_{n}^{2}$; Δf and Δθ represent carrier frequency offset and phase offset, respectively.

Usually, in binary hypothesis problems, it is necessary to set a reliable judgment threshold based on certain judgment criteria to determine whether $H_{0}$ holds or $H_{1}$ holds. Common criteria include the maximum a posteriori estimation criterion, the minimum probability of miscarriage of justice criterion, etc. When the received signal exceeds the threshold value, it is determined that the signal exists. On the contrary, it is determined that the signal does not exist. The minimum probability of misclassification criterion is to choose a method that minimizes the probability of misclassification, thereby making the judgment results more accurate. The discriminant results of the binary hypothesis include four types, as shown in Table 1.

The probability $P(H_{i}/H_{j})$ corresponding to the decision result $\left(H_{i}/H_{j}\right)$ represents the probability that the decision result is $H_{i}$ if $H_{j}$ is true. Among them, $P(H_{1}/H_{0})$ is defined as the false alarm probability, $P(H_{0}/H_{1})$ is defined as the missed detection probability, and $P(H_{1}/H_{1})$ is defined as the detection probability. $P(H_{0}/H_{0})$ represents the probability that the judgment result is $H_{0}$ when $H_{0}$ is true, and there is no more specific definition for it. In the training process of the model, we evaluated the values of $P(H_{0}/H_{0})$ plus $P(H_{1}/H_{1})$, but, in the estimation performance analysis process, we did not care about the indicator $P(H_{0}/H_{0})$, and we focused more on the results of $P(H_{1}/H_{1})$, which is the detection probability.

This paper transforms the DSSS signal detection problem into a binary classification problem using deep learning to replace threshold discrimination with softmax classifiers to achieve DSSS signal detection and measuring the detection performance of the model’s proposed method through detection probability.

## 3. Principle of Proposed DSSS Signal Detection Based on CNN

**Figure 2:**
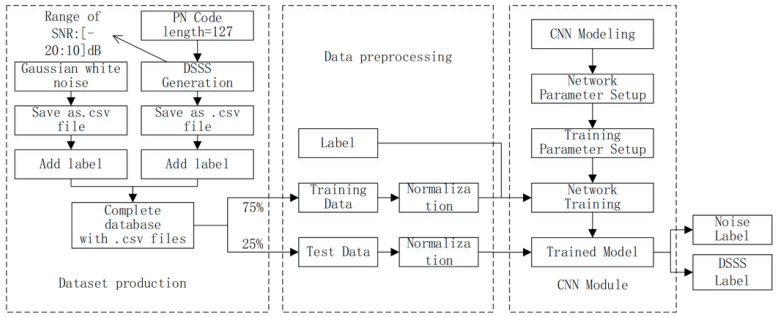
Overall block diagram of DSSS signal detection based on CNN.

The detection of DSSS signals based on CNN mainly includes several parts: dataset production, data preprocessing, CNN network model building, model training, and prediction. The classification of signals and noise is achieved through the softmax layer of the network. The feature data output through multiple convolutional layers is fed into the softmax layer, and the confidence level of the corresponding category is output. The category with the highest confidence level is the detection result. Finally, the trained model can detect other untrained unknown signals, as shown in Figure 2.

### 3.1. Data Preprocessing

The network model in this article is built under the Tensorflow framework as the input data requirement for the convolutional layer is 3D data of M×N×P; M×N represents the size of input data, and *P* represents the number of input channels. Therefore, first separate and store the data from the I/Q channels in the simulation-generated dataset into two .csv files, read them separately, and use the np.array ([I_data,Q_data]) function to combine them into the format of 2×M×N, where 2 represents the number of channels, so we also need to use the swataxes() function to change the format of the dataset from 2×M×N to M×N×2. In this way, the input format of the dataset is the same as the input requirements of the network. Secondly, in order to reduce the impact of signal power on the model and speed up the training and rate of convergence of the network, the input data shall be normalized before being input into the CNN network.

Secondly, in order to reduce the impact of signal power on the model and speed up the training and rate of convergence of the network, the input data shall be normalized before being input into the CNN network using Equation (6).

(6) $$\hat{r}\left(k\right)=\frac{r(k)}{\frac{1}{L_{1}}\sum_{k=0}^{L_{1}-1}{\left|r\left(k\right)\right|}^{2}}$$

In the above equation, r(k) represents the received signal, and $L_{1}$ represents the length of the received signal.

### 3.2. Network Model and Algorithm Reasoning

**Figure 3:**
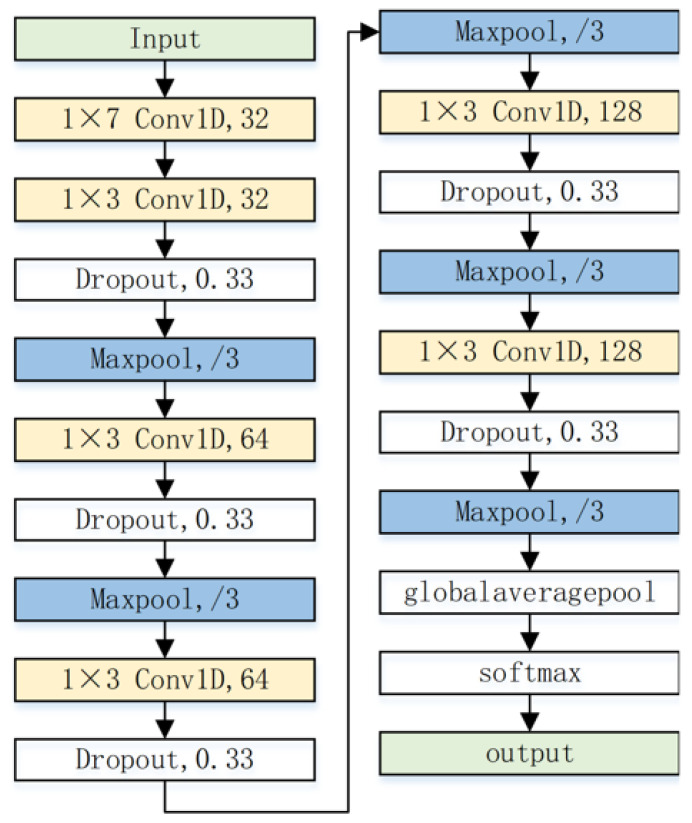
CNN structure diagram.

The CNN network designed in this article consists of 6 convolutional layers, 5 maximum pooling layers, 1 global pool averaging layer, and 1 softmax layer. The network structure is shown in Figure 3.

In the above figure, “m×n Conv1D,c,/f” indicates that the convolutional kernel size used in this one-dimensional convolutional layer is m×n. The number of channels is *c*, the downsampling factor is *f*, and the parameters of the network model are shown in Table 2.

Assuming the training set of the network is shown in Equation (7)

(7) $$ℜ=\{\left(r_{train}^{\left(1\right)},l_{train}^{\left(1\right)}\right),\left(r_{train}^{\left(2\right)},l_{train}^{\left(2\right)}\right),\ldots ,\left(r_{train}^{\left(n\right)},l_{train}^{\left(n\right)}\right)\}$$

where $r_{train}^{\left(i\right)}$ represents the *i*-th signal sample, with a size of 1×2048×2, $l_{train}^{\left(i\right)}$ represents the true label of the *i*-th sample. The activation function in this paper selects ReLu function. After passing through multiple convolution layers and pooling layers, the new feature is expressed as $Y=[y_{1},y_{2},\ldots ,y_{N}]$, and, after passing through the global average pooling layer, the output is shown in Equation (8).

(8) $$z\left(y\right)=\frac{1}{N}\sum_{j=0}^{N-1}y_{j}$$

Finally, input the output of the global average pooling layer into the softmax layer and calculate the confidence levels $f(z_{n})$ of the input signal belonging to DSSS signal and noise signal according to Equation (9).

(9) $$p\left(l_{train}^{\left(i\right)}=n|r_{train}^{\left(i\right)},\theta \right)=\frac{e^{\theta_{n}^{T}r_{train}^{\left(i\right)}}}{\sum_{l=1}^{N}e^{\theta_{n}^{T}r_{train}^{\left(l\right)}}}$$

The label corresponding to the maximum confidence level ${\hat{l}}_{train}^{\left(i\right)}$ is the estimation result. In the training process, Adam algorithm is selected as the optimization algorithm to minimize the loss function, and cross-entropy is selected as the error loss function. Then, the network parameters are updated as Equation (10) [17].

(10) $$\theta_{n}=\theta_{n-1}-\eta \frac{{\hat{s}}_{n}}{\sqrt{{\hat{v}}_{n}}+\varepsilon }$$

where θ is the vector composed of the weights and deviations of the network, η is the learning rate, ${\hat{s}}_{n}$ and ${\hat{v}}_{n}$ are the first-order moment and the deviation from the second-order moment, respectively, and ε is a small constant, aimed at increasing the stability of the algorithm.

## 4. Simulation Experiment and Result Analysis

### 4.1. Dataset Production

First, 17-bit information code is randomly generated, and then the original information sequence is spread-spectrum-modulated using a 127 m sequence, and, finally, the spread spectrum signal is modulated using the BPSK modulation method to obtain a direct sequence spread spectrum signal, with a spread spectrum code rate of 1 Mbps and sampling rate of 2 Mbps. The length of each signal obtained after sampling is 2048, and the actual and imaginary parts are the input data of the I and Q channels. The I/Q channels are used as the channel dimensions of the dataset. Therefore, the size of a single sample in the dataset is 1×2048×2. The range of signal-to-noise ratio is [−20:2:10] dB. Further, 1000 DSSS sample signals are generated under each signal-to-noise ratio, and then noise signals of the same size are generated. They are combined to form a complete dataset, which includes DSSS signals under different signal-to-noise ratio conditions, allowing the trained single network to adapt to different signal-to-noise ratios. Before training, the entire dataset needs to be unordered, and then 75% of it needs to be taken as the training dataset and 25% as the testing dataset.

### 4.2. Training Environment and Process

**Figure 4:**
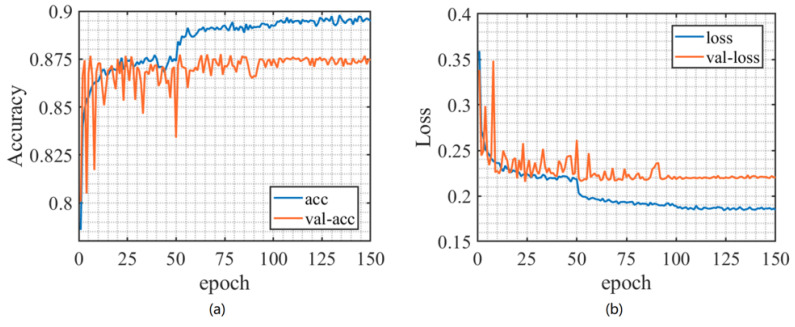
Training process: (**a**) accuracy curve, (**b**) loss function curve.

The model training was completed on a computer with a CPU model of Intel(R) Core(TM)i7-10875H@2.30 GHz and a GPU model of NVIDIA GeForce RTX 2060 6 GB, with a running memory of 32 GB. The training parameters of the CNN network are shown in Table 3. In the training process, the change in the accuracy and loss function is shown in Figure 4, where acc and loss, respectively, represent the accuracy and loss function; val of the training set in the model training process_Acc and val_Loss indicate the correctness rate and loss function of the test set, respectively. Observing Figure 4b, it was found that loss no longer decreased after training 100 epochs. At the same time, observing Figure 4a, it can be observed that, after training 100 epochs, the accuracy of the training and testing sets tends to stabilize and no longer increases. In the end, the accuracy rate of the training set was 89.49%, while the accuracy rate of the test set was 87.46%. It is worth mentioning that, during the training process, the signal-to-noise ratio range of the training set signal we used is from −20 dB to 10 dB. Although the large signal-to-noise ratio range affected the overall accuracy to some extent, it also improved the generalization ability of the model.

### 4.3. Estimation Performance Analysis under Different Signal-to-Noise Ratios

**Figure 5:**
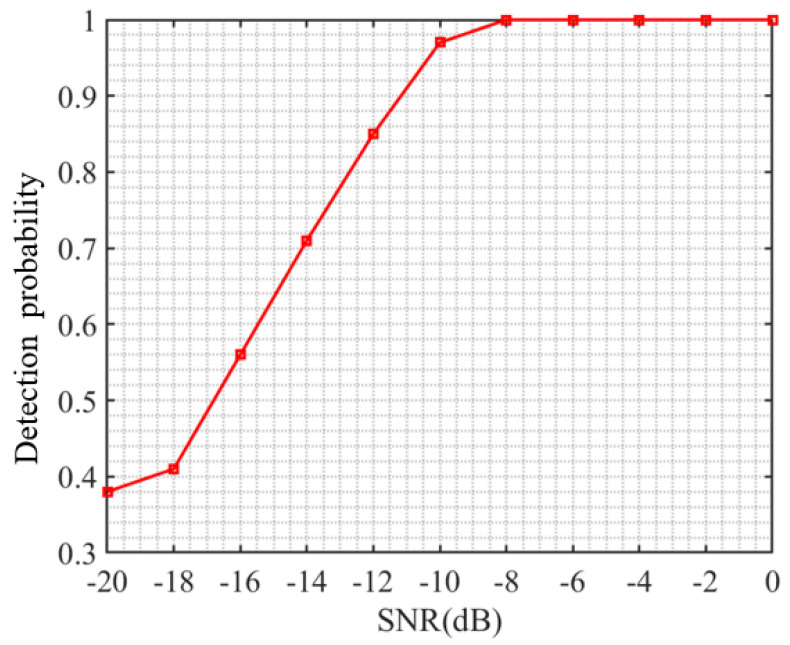
Detection probability curves under different signal-to-noise ratios.

This section verifies the detection performance of the CNN-based DSSS signal detection method proposed in this paper under different signal-to-noise ratio conditions. The generation method of the test dataset is the same as that of the dataset in Section 4.1, with 100 test signals generated for each signal-to-noise ratio. The newly generated data are tested using the trained model, and the results are shown in Figure 5. From the figure, it can be seen that the detection probability of the proposed method for DSSS signals continues to increase with the increase in signal-to-noise ratio, reaching a detection probability of 1 at −8 dB. It can also be observed that, when the detection probability of the method in this paper is below −10 dB, the detection probability decreases faster with the signal-to-noise ratio, indicating that the detection performance of the method in this paper decays faster when it is below −10 dB.

### 4.4. Estimation Performance Analysis under Different Spread Spectrum Code Lengths

**Figure 6:**
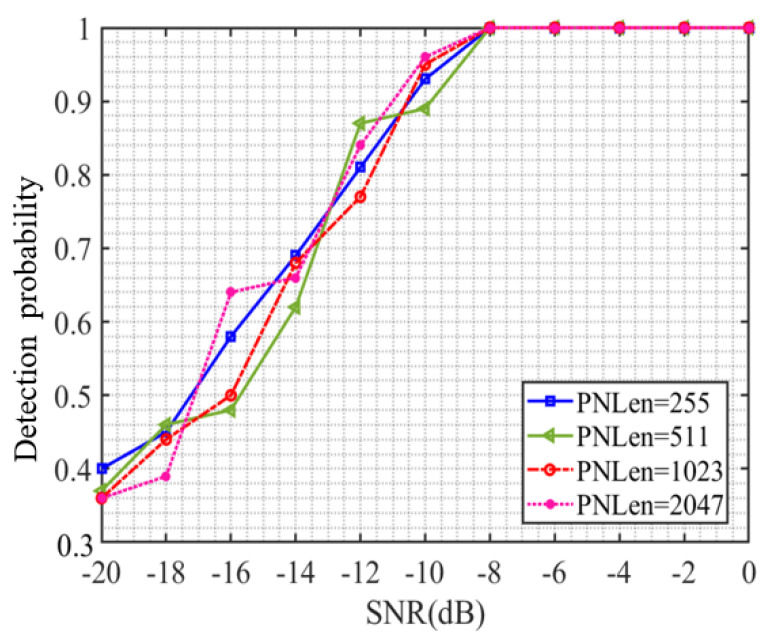
Detection probability curve under different spread spectrum code lengths.

The previous experiment verified the detection performance of the CNN model proposed in this article for DSSS signals under different signal-to-noise ratios. In order to verify the generalization ability of the CNN model proposed in this article, this section used a trained CNN model to detect DSSS signals with spreading code lengths of 255, 511, 1023, and 2048 under different signal-to-noise ratios. The results are shown in Figure 6, where PNLen represents the length of the selected spreading code. From Figure 6, it can be seen that the detection probability continuously increases with the increase in signal-to-noise ratio. When the signal-to-noise ratio reaches −8 dB, the detection probability of DSSS signals generated by using different lengths of spread spectrum codes reaches 1, which is the same as the detection performance when the spread spectrum code length is 127. When the length of the spread spectrum code used for DSSS signals is below −8 dB, the detection probability varies. Under the same signal-to-noise ratio, the maximum difference in detection probability is 0.16. However, there is no obvious relationship between the detection probability and the length of the spread spectrum code under the same signal-to-noise ratio. Therefore, the detection performance of the CNN model in this paper is not affected by the length of the spread spectrum code used for DSSS signals and has a certain degree of generalization ability.

### 4.5. Estimation Performance Analysis under QPSK Modulation

**Figure 7:**
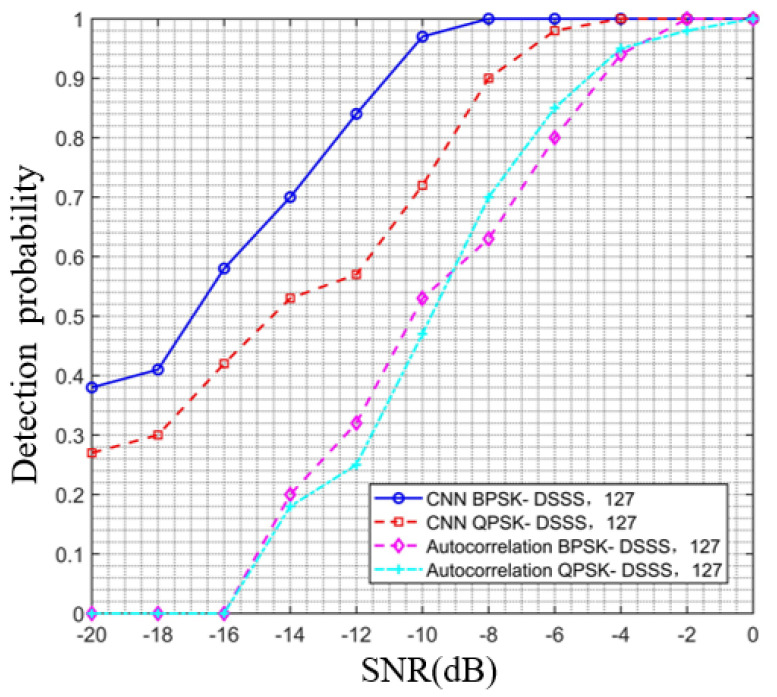
Comparison curve of detection probability between QPSK- and BPSK-modulated DSSS signals using the proposed algorithm and traditional autocorrelation algorithm.

**Figure 8:**
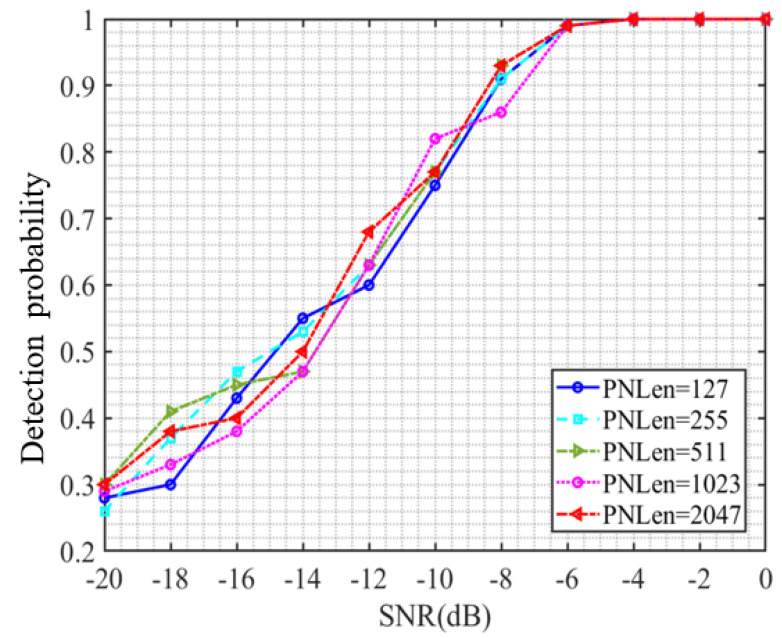
Detection probability curve during QPSK modulation.

This section verifies and analyzes the detection performance of the CNN model proposed in this article on QPSK-modulated DSSS signals (abbreviated as QPSK-DSSS) under different signal-to-noise ratios, and the results are shown in Figure 7 and Figure 8. Figure 7 shows the comparison curve of the detection probabilities of the QPSK-DSSS signal and BPSK-DSSS signal with a spreading code length of 127 under different signal-to-noise ratios using the proposed algorithm and traditional autocorrelation algorithm. Observing Figure 7, it can be observed that the detection probability of the QPSK-modulated DSSS signal reaches 1 at −4 dB, while the detection probability of the BPSK-modulated DSSS signal reaches 1 at −8 dB, indicating that the detection performance of the model in this paper for QPSK-DSSS signals is reduced by 4 dB compared to the BPSK-DSSS signal. In addition, comparing the results of our algorithm with traditional autocorrelation algorithms, it can be found that, when the signal-to-noise ratio is below −16 dB, the detection probability of traditional autocorrelation algorithms is 0, and, when the signal-to-noise ratio approaches 0 dB, the detection probability is 1, indicating significantly lower detection performance than our model.

Figure 8 shows the curve of the detection probability of QPSK-DSSS signals with different lengths of spread spectrum codes as a function of signal-to-noise ratio, where PNLen represents the length of the selected spread spectrum code. Observing the detection probability of QPSK-modulated DSSS signals using different lengths of spread spectrum codes, it can be found that, after the signal-to-noise ratio reaches −6 dB, the detection probability is completely unaffected by the length of the spread spectrum code used. When the noise level is below −6 dB, the detection probability of QPSK-DSSS signals with different spreading code lengths under the same signal-to-noise ratio conditions is not entirely the same in this model. However, no obvious pattern was found when observing the relationship between detection probability and spreading code length. Therefore, this model is still applicable to QPSK-DSSS signals, and the detection performance is only related to the signal-to-noise ratio and almost independent of the spreading code length. However, the overall detection performance is lower than that of BPSK-DSSS signals.

### 4.6. Estimation Performance Analysis of Spread Spectrum Codes Using Gold Sequences

**Figure 9:**
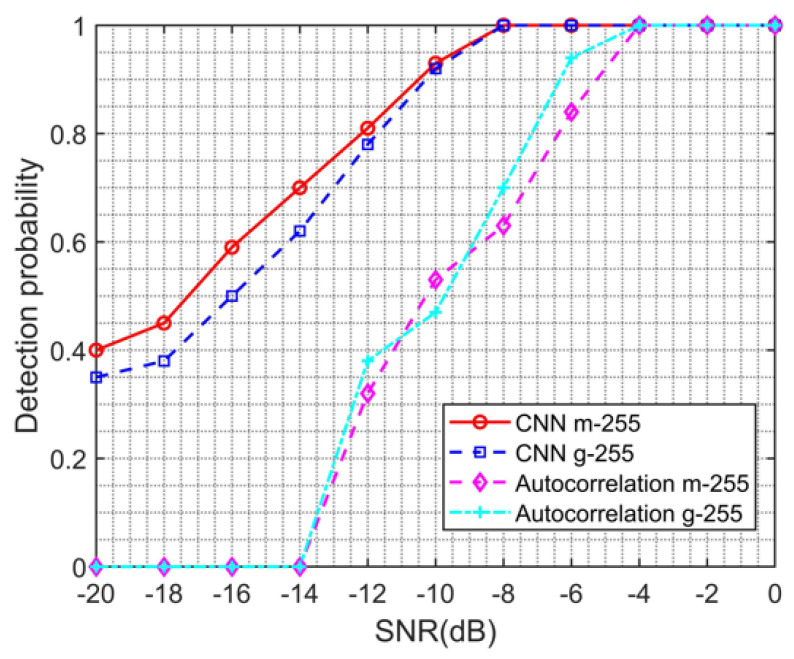
Detection probability curve of spread spectrum code using m-sequence and Gold sequence of the same length using the proposed algorithm and traditional autocorrelation algorithm.

**Figure 10:**
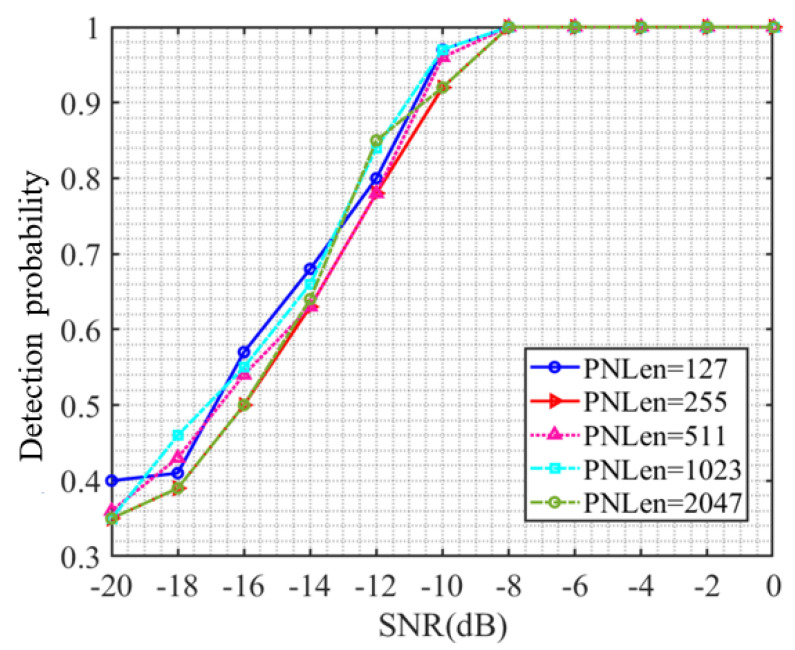
Detection probability curve of spread spectrum code using Gold sequences of different lengths.

The Gold sequence is a pseudo-random sequence with good characteristics proposed and analyzed by R. Gold in 1967 based on the m-sequence. It is composed of two optimum pairs of m-sequences with equal code length and the same code clock rate, which is added by modulo 2. Gold code sequence is a code sequence based on the m-sequence, which has excellent autocorrelation and cross-correlation characteristics and generates a large number of sequences. In this section, the Gold sequence was selected as the spreading code. Under each signal-to-noise ratio, 100 DSSS signals of different lengths of spreading codes were generated. The trained CNN model was used to detect them, and the results are shown in Figure 9 and Figure 10. Figure 9 shows a comparison of the detection probabilities of the proposed algorithm and the traditional autocorrelation algorithm for DSSS signals with a spreading code length of 255, using the m sequence and the Gold sequence, respectively. Observing Figure 9, it can be observed that, regardless of using the m sequence or the Gold sequence for spread spectrum modulation, the detection probability reaches 1 at −8 dB, which means that the DSSS signal can be accurately detected at −8 dB. When the signal-to-noise ratio is below −8 dB, the detection probability of DSSS signals using the Gold sequence is lower than that of DSSS signals using the m sequence, with a maximum difference of 0.08. In summary, it indicates that the model proposed in this paper is also applicable to DSSS signals using Gold sequences for spread spectrum modulation. However, when the signal-to-noise ratio is below −8 dB, the detection probability of DSSS signals using Gold sequences decreases faster. In addition, comparing the results of our algorithm with traditional autocorrelation algorithms, it can be found that, when the signal-to-noise ratio is below −14 dB, the detection probability of the traditional autocorrelation algorithm for DSSS signals using both Gold and m sequences is 0, and, only when the signal-to-noise ratio is greater than −4 dB, the detection probability is 1, which is 4 dB different from our model.

Figure 10 shows the detection probability of DSSS signals using Gold sequences of different lengths as a function of signal-to-noise ratio. From the figure, it can be seen that, regardless of the length of the spread spectrum code sequence selected in the DSSS signal, the detection probability of the DSSS signal reaches 1 at −8 dB, indicating that the model in this paper is completely unaffected by the length of the spread spectrum code above −8 dB for DSSS signals modulated by Gold sequences. When the length of the Gold sequence is below −8 dB, the detection probability varies. At the same signal-to-noise ratio, the maximum difference between the maximum and minimum detection probabilities is 0.07. However, there is no significant relationship between the fluctuation and the length of the spread spectrum code. This indicates that the performance of the model in this paper is unstable when detecting DSSS signals below −8 dB, but the fluctuation of the detection probability does not exceed 0.07. From Figure 10, it can also be observed that the detection probability of the DSSS signal using the Gold sequence as the spreading code sequence decreases faster when it is below −10 dB, which is consistent with the results when using the m sequence as the spreading code sequence. This indicates that the detection performance of the model in this paper decays faster when it is below −10 dB.

### 4.7. Comparative Analysis of Different Methods

**Figure 11:**
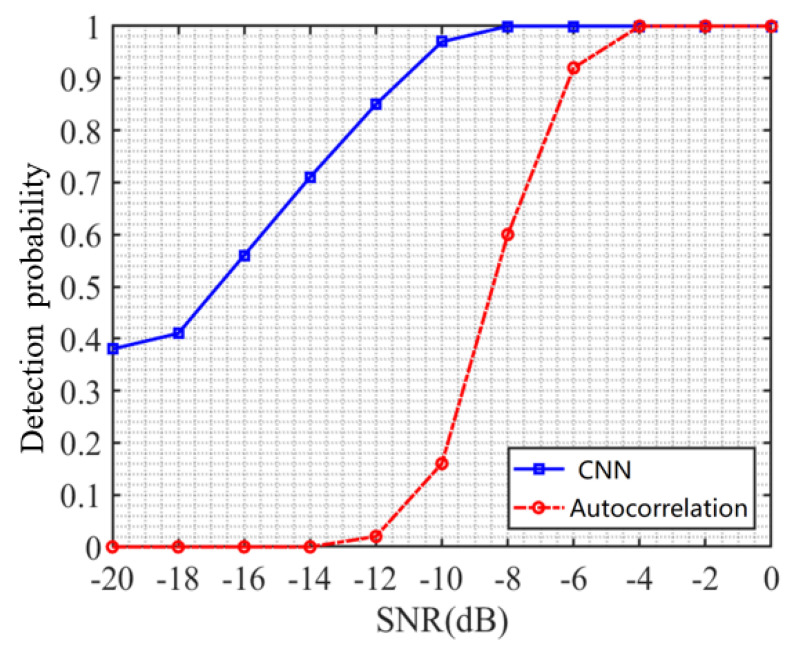
Detection probability curve using different methods.

This section compares the CNN-based DSSS signal detection method proposed in this article with traditional autocorrelation detection methods, and the results are shown in Figure 11. From the figure, it can be seen that, at −14 dB, the traditional autocorrelation detection method has completely failed and cannot detect DSSS signals. However, the detection probability of the method proposed in this paper is 0.71 at −14 dB, indicating that the detection performance of the method in this paper is much higher than that of the autocorrelation detection algorithm in low signal-to-noise ratio situations. Moreover, from Figure 11, it can be observed that the detection probability of the autocorrelation detection method only reaches 1 at −4 dB, while the detection probability of the method proposed in this paper has already reached 1 at −8 dB. Therefore, compared to the autocorrelation detection method, the overall performance gain of the CNN-based detection method proposed in this paper is 4 dB.

## 5. Conclusions

In order to solve the problem of insufficient intelligence in electronic reconnaissance technology in modern electronic warfare, this paper conducted extensive research on signal detection in electronic reconnaissance technology and attempted to apply the favored neural network model to DSSS signals detection.

In this paper, deep learning technology is introduced into DSSS signal detection, and a six-layer CNN network is designed to realize the detection of DSSS signals. We model the presence detection of DSSS signals as a binary classification problem with a CNN network. During network training, the I/Q data of standard DSSS signals are directly input into the CNN model, and appropriate network parameters are set, and the respective characteristics of DSSS signals and noise signals are automatically obtained after training. The training dataset contains the BPSK-modulated DSSS signal with a spreading code length of 127 and a signal-to-noise ratio (SNR) from −20 dB to 10 dB, which has a large range of SNR. The signal to be tested is input into the trained network for detection. The experimental results show that the detection probability of this method reaches 100% at −8 dB, which improves the overall performance by 4 dB compared with the traditional autocorrelation detection method. It is also verified that the DSSS signal uses different spreading code lengths, QPSK modulation, and Gold sequence. The model is still applicable and has good experimental results, which shows that the model has good robustness.

In the future, we will attempt to use neural network models with better performance for more precise signal recognition and parameter estimation, such as spreading code period estimation.

## Figures and Tables

**Table 1:** Binary hypothesis judgment result.

Judgment	Hypothesis	
	$H_{0}$	$H_{1}$
$H_{0}$	($H_{0}/H_{0}$)	($H_{0}/H_{1}$)
$H_{1}$	($H_{1}/H_{0}$)	($H_{1}/H_{1}$)

**Table 2:** CNN network model parameters.

Network Layer Name	Dimension	Network Layer Name	Dimension
Input	1×2048×2	MaxPooling1D	1×151×64
Conv1D	1×4096×32	Conv1D	1×151×128
Conv1D	1×4096×32	Dropout	1×151×128
Dropout	1×4096×32	MaxPooling1D	1×50×128
MaxPooling1D	1×1365×32	Conv1D	1×50×128
Conv1D	1×1365×64	Dropout	1×50×128
Dropout	1×1365×64	MaxPooling1D	1×16×128
MaxPooling1D	1×455×64	Globalaveragepool	1×1×128
Conv1D	1×455×64	Softmax	1×1×2
Dropout	1×455×64		

**Table 3:** CNN model training parameters.

Parameter	Parameter Value
Initial learning rate	0.001
Training rounds	150
Small batch size	8
Learning rate decline cycle	50
Learning rate decline coefficient	0.1
Droupout Rate	0.33
Optimizer	Adam

## Data Availability

The study did not report any data.
